# Female Type 1 Diabetic Akita Mice Demonstrate Increased Bladder Contractility via FP Receptor Activation due to NLRP3-Mediated Inflammation

**DOI:** 10.31083/j.fbl2904154

**Published:** 2024-04-18

**Authors:** Michael R. Odom, Francis M. Hughes, NiQuava Pope, Huixia Jin, J. Todd Purves

**Affiliations:** 1 Department of Urology, Duke University School of Medicine, Durham, NC 27710, USA

**Keywords:** NLRP3, diabetic bladder dysfunction, Akita, Type 1 diabetes, prostaglandin, FP receptor, inflammation, myograph

## Abstract

**Background:**

Diabetic bladder dysfunction (DBD) is driven in part by inflammation which dysregulates prostaglandin release in the bladder. Precise inflammatory mechanisms responsible for such dysregulation have been elusive. Since prostaglandins impact bladder contractility, elucidating these mechanisms may yield potential therapeutic targets for DBD. In female Type 1 diabetic Akita mice, inflammation mediated by the nucleotide-binding domain, leucine-rich–containing family, pyrin domain–containing-3 (NLRP3) inflammasome is responsible for DBD. Here, we utilized female Akita mice crossbred with *NLRP3* knock-out mice to determine how NLRP3-driven inflammation impacts prostaglandin release within the bladder and prostaglandin-mediated bladder contractions.

**Methods:**

Akita mice were crossbred with *NLRP3*^−/−^ mice to yield four groups of non-diabetics and diabetics with and without the *NLRP3* gene. Females were aged to 30 weeks when Akitas typically exhibit DBD. Urothelia and detrusors were stretched *ex vivo* to release prostaglandins. Prostaglandin E2 (PGE2) and prostaglandin F2*α* (PGF2*α*) were quantified using enzyme linked immunosorbent assays (ELISA). In separate samples, *ex vivo* contractile force to PGE2 and PGF2*α* +/− the prostaglandin F (FP) receptor antagonist, AL8810, was measured. FP receptor protein expression was determined via western blotting.

**Results:**

Stretch-induced PGE2 release increases in urothelia but decreases in detrusors of diabetics. However, PGE2-mediated bladder contractions are not impacted. Conversely, diabetics show no changes in PGF2*α* release, but PGF2*α*-mediated contractions increase significantly. This is likely due to signaling through the FP receptors as FP receptor antagonism prevents this increase and diabetics demonstrate a four-fold increase in FP receptor proteins. Without NLRP3-mediated inflammation, changes in prostaglandin release, contractility, and receptor expression do not occur.

**Conclusion:**

NLRP3-dependent inflammation dysregulates prostaglandin release and prostaglandin-mediated bladder contractions in diabetic female Akita mice via FP receptor upregulation.

## 1. Introduction

Diabetic bladder dysfunction (DBD) develops in half of all patients with diabetes [1–3]. Their symptoms are variable with underactive bladder (UAB) being one of the more bothersome phenotypes. Patients may experience a decreased sensation of bladder fullness, slow urine stream, and overflow incontinence indicative of hypocontractile bladders and often rely on self-catheterization to void, since no targeted pharmacological therapies are available. In order to develop much-needed therapies, further elucidation of the mechanisms responsible for DBD are warranted.

A hallmark of diabetes is low-grade inflammation mediated by a host of pro-inflammatory immune responses [4–6]. Of particular interest is inflammation mediated by the nucleotide-binding domain, leucine-rich–containing family, pyrin domain–containing-3 (NLRP3) inflammasome. This component of the innate immune system is highly expressed in bladder urothelial cells. Diabetic metabolites, such as uric acid and high-mobility group box 1 protein, activate NLRP3 to release pro-inflammatory cytokines [7]. Notably, we have shown NLRP3 to be a critical inflammatory pathway responsible for DBD in female type 1 diabetic Akita mice [7,8]. Despite its critical importance, it is unclear what downstream signaling pathways are dysregulated by NLRP3 activation in the bladder. However, in the kidney, NLRP3 activation within murine proximal tubule cells increases prostaglandin production [9,10], suggesting similar pathways may be active in the bladder.

Prostaglandins are eicosanoids derived from fatty acid synthesis via cyclooxygenases and they are produced throughout the body, including urothelia and bladder smooth muscle [11,12]. Inflammatory conditions such as diabetes have been shown to upregulate prostaglandin production and release [4,13–15]. Once released, prostaglandins activate receptors in an autocrine and paracrine manner within close proximity from where they are released and are quickly degraded into metabolites which accumulate in urine. Such metabolites are upregulated in patients with either type 1 or type 2 diabetes [4,13–15]. Moreover, controlling blood glucose also reduces the levels of prostaglandin metabolites in type 1 diabetic urine [14]. Despite clinical evidence of increased prostaglandin production in diabetic bladders, the physiological effects of prostaglandins on diabetic bladder function are not well understood.

Several prostaglandins have been the focus of scientific inquiry in the diabetic bladder - particularly prostaglandin E2 (PGE2) and prostaglandin F2*α* (PGF2*α*) [16–18]. These prostaglandins, as well as their respective metabolites, exert potent pro-contractile effects on bladder smooth muscle (detrusor) in humans, macaques, pigs, and rodents by activating two primary classes of membrane bound prostaglandin receptors [19–21]. PGE2 has a higher affinity for the EP class of receptors while PGF2*α* has a higher affinity for prostaglandin F (FP) receptors. In preclinical models, diabetes has been shown to dysregulate bladder contractility to PGE2 and PGF2*α* [16]. However, it remains unclear how diabetes impacts populations of prostaglandin receptors responsible for these effects. Here, we determine the role of NLRP3-mediated inflammation associated with diabetes in regulating prostaglandin signaling pathways responsible for bladder contractions using the diabetic Akita mouse model of UAB and Akita mice lacking the *NLRP3* gene which fail to develop DBD [8].

## 2. Methods

### 2.1 Animals

Animal procedures were conducted in accordance to guidelines set forth by the National Institutes of Health Guide for the Care and Use of Laboratory Animals and approved by the Duke University Medical Center Institutional Animal Care and Use Committee (approval number: A088–22-05). Type 1 diabetic Akita mice (C57BL/6J-Ins2Akita/J; stock #003548; Jackson Laboratory, Bar Harbor, ME, USA) have a heterozygous mutation of the insulin 2 (*Ins2*) gene which causes them to spontaneously develop hyperglycemia by 4–5 weeks of age. To determine the specific role of NLRP3 in the progression of diabetes, Akita mice and *NLRP3*^−/−^ (B6.129S6-Nlrp3^tm1Bhk^/J; stock #021302) mice were purchased from Jackson Laboratory and used as founding mice. The origin strain of the *NLRP3*^−/−^ mice (129S6/SvEvTac) is different from the Akita background (C57BL/6J), but these mice were backcrossed to C57BL/6J for *>*11 generations prior to purchase. The Duke University Breeding Core Facility bred all mice colonies. Mice were co-housed at 22 ± 2 °C on 12 hour light/dark cycles. Mice were given rodent chow and water *ad libitum*. Mice were genotyped by Transnetyx, Inc. (Cordova, TN, USA) and provided to the laboratory at 4 weeks of age. Genotype results placed animals into one of four different experimental groups described below. Female mice were used in this study and aged to 30 weeks – a time point at which female Akita mice develop UAB [8].

1. *Non-diabetic NLRP3*^+/+^: normal *Ins2* gene; normal *NLRP3* gene.
2. *Diabetic NLRP3*^+/+^: heterozygous *Ins2* gene mutation; normal *NLRP3* gene.
3. *Non-diabetic NLRP3*^−/−^: normal *Ins2* gene; *NLRP3* gene deletion.
4. *Diabetic NLRP3*^−/−^: heterozygous *Ins2* gene mutation; *NLRP3* gene deletion.

### 2.2 Blood Glucose

Blood obtained from the lateral tail vein was collected to measure blood glucose using the AimStrip Plus glucometer and testing strips (Lot number 396051.Germaine Laboratories, San Antonio, TX, USA).

### 2.3 Ex Vivo Prostaglandin Release

Bladders were excised and immediately put in ice cold Krebs solution made up of 118.5 mM NaCl, 58.44 mM KCl, 1.2 mM MgCl_2_, 23.8 mM NaHCO_3_, 1.2 mM KH_2_PO_4_, 11 mM dextrose and 1.8 mM CaCl_2_ in distilled water. The dome and trigone were dissected away and 2 mm wide strips of bladder were prepared for experimentation. The urothelia-lined mucosa and detrusor from each 2 mm wide strip were carefully separated and mounted into separate myograph chambers (820M Danish Myograph Technology, Aarhus, Denmark) containing aerated (95% O_2_ and 5% CO_2_) Krebs solution maintained at 37 °C. Tissue was allowed to acclimate for 30 minutes. After the acclimation period, the Krebs solution was exchanged and a 3 mN stretch was applied to tissues and maintained for 10 minutes in a manner similar to existing methodology which we modified to match the basal stretch conditions used for *ex vivo* bladder contractility experiments [22]. The solution was then collected for prostaglandin analysis and stored at −80 °C. The amount of released PGE2 and PGF2*α* was quantified using commercially available ELISA kits as directed by the manufacturer: Prostaglandin E2 Parameter Assay Kit (R&D Systems, Minneapolis, MN, USA; item #KGE004B) and PGF2 alpha High Sensitivity ELISA kit (Abcam, Cambridge, UK; item #ab133056).

### 2.4 Ex Vivo Bladder Contractility

Bladders were excised and placed in ice cold Krebs solution consisting of 118.5 mM NaCl, 58.44 mM KCl, 1.2 mM MgCl_2_, 23.8 mM NaHCO_3_, 1.2 mM KH_2_PO_4_, 11 mM dextrose and 1.8 mM CaCl_2_ in distilled water. The dome and trigone were dissected away and 2 mm wide strips of bladder with an intact mucosa and urothelia were prepared for experimentation. Bladder strips were mounted in myographs (820M Danish Myograph Technology, Aarhus, Denmark) containing aerated (95% O_2_ and 5% CO_2_) Krebs solution maintained at 37 °C. Tissue was allowed to acclimate for 30 minutes. A basal tension of 3 mN was maintained on all tissue for an additional 30 minutes. Krebs solution was replaced every 15 minutes except during maintenance of resting tension and incubation of pharmacological antagonists. Contractile responses to a Krebs solution containing 120 mM KCl verified tissue viability and contractile force generated by all subsequent agonists were normalized to this value. Data acquisition was achieved using a PowerLab 4/26 system (ADInstruments, Colorado Springs, CO, USA) and accompanying LabChart Pro version 8 software (ADInstruments, Colorado Springs, CO, USA).

Stock solutions of PGE2 (Cayman Chemical Co.; item #14010), PGF2*α* (Cayman Chemical Co., Ann Arbor, MI, USA; item #16010), and the FP receptor antagonist, AL8810 (Cayman Chemical Co.; item #16735) were dissolved in ethanol at 10^−1^ M concentrations and stored at −20 °C. Subsequent dilutions were prepared in distilled water on the day of experiments. Increasing cumulative concentrations of PGE2 and PGF2*α* were administered at 3-minute intervals to construct concentration response curves of each agonist. At each concentration, contractile force plateaued within the 3-minute interval. The same tissues were then incubated witph a 10^−5^ M concentration of the partial FP receptor antagonist, AL8810, for 30 minutes. Then, the PGF2*α* concentration response curve was repeated in the presence of AL8810.

### 2.5 Protein Expression

At 30 weeks, mice were euthanized and bladders were removed. The detrusor was carefully separated from the mucosa and stored at −80 °C. Detrusors were homogenized in ice cold RIPA buffer (150 mM NaCl, 1% Triton X-100, 0.5% sodium deoxycholate, 0.1% SDS, and 50 mM Tris; pH 7.4) supplemented with a protease and phosphatase inhibitor cocktail at 1× concentration (Halt; Thermo Fisher, Waltham, MA, USA). Homogenates were centrifuged at 16,000 ×g for 15 minutes to remove cellular debris. Supernatant was collected and protein concentrations of these supernatants were determined using a bicinchoninic acid (BCA) protein assay as directed by manufacturer (Pierce BCA Assay; Thermo Fisher).

Proteins (100 μg) from each detrusor were reduced in lithium dodecyl sulfate sample buffer and heated at 70 °C for 5 minutes. Reduced proteins were then loaded into two separate 4–12% Bis-Tris SDS polyacrylamide gels (Invitrogen, Waltham, MA, USA) with each gel containing 50 μg of protein per detrusor. Both gels containing the molecular weight marker, Precision Plus Protein WesternC Blotting Standard (Bio-Rad; Hercules, CA, USA), underwent simultaneous electrophoresis in a duel chamber tank containing 1× MES SDS Running buffer (NuPage; Invitrogen) at a constant 80V for 2.5 hours. Both gels were then transferred onto the same nitrocellulose membrane (0.2 μm pore size) using the iBlot 2 semi-dry transfer apparatus (8 minutes, 20V; Invitrogen). Membranes were then cut to reflect the two separate gels containing identically prepared protein samples. Both membranes were blocked at room temperature for 1 hour in phosphate buffered saline (PBS) containing 5% bovine serum albumin.

Both membranes were probed simultaneously overnight at 4 °C in PBS containing 5% bovine serum albumin, 0.1% Tween 20, and either a primary antibody or primary antibody plus a blocking peptide. The first membrane was probed with a primary antibody against the FP receptor (1:200; Cayman Chemical Co.; item #101802) while the second membrane was probed with a premixed solution containing the FP receptor primary antibody (1:200) and a FP receptor blocking peptide (1:100; Cayman item #301802). Following the overnight primary antibody +/− blocking peptide incubation, secondary antibody incubation was performed at room temperature for 1 hour using a goat anti-rabbit alexa fluor 488 antibody (1:5,000; Jackson Immunoresearch item #111–545-144) in PBS containing 5% bovine serum albumin and 0.1% Tween 20. Labeled proteins were detected using the ChemiDoc MP system (Bio-Rad). Membranes were re-probed with a primary antibody against the housekeeping protein, GAPDH (1:5000; GeneTex item #GTX100118, Irvine, CA, USA). Protein expression analysis was conducted using Image Lab 6.1 software (Bio-Rad) and target protein expression was normalized to GAPDH expression. Representative western blot images were converted from color images to gray scale using ImageJ software (National Institutes of Health, Bethesda, MD, USA). Uncropped western blots used for data analysis are provided as Supplementary Figs. 1,2,3 section.

### 2.6 Statistical Analysis

Prism 9 software (GraphPad; San Diego, CA, USA) was used to create all graphs and perform statistical analysis. All data is reported as mean ± standard error of the mean. For protein expression and *ex vivo* prostaglandin release analysis, one-way analysis of variances (ANOVA) with Tukey post hoc tests were performed. For *ex vivo* bladder contractility analysis of concentration response curves, two-way ANOVAs with Bonferroni post hoc tests were performed. Concentration response curves were fitted to a logistic function by nonlinear regression and the −log half maximal effective concentration (EC_50_) was calculated using Prism 9 software. Statistical significance is defined as *p* < 0.05. Results of these statistical analyses are reported in the manuscript body.

## 3. Results

### 3.1 NLRP3 Gene Deletion Has No Impact on Blood Glucose

**Fig. 1.**
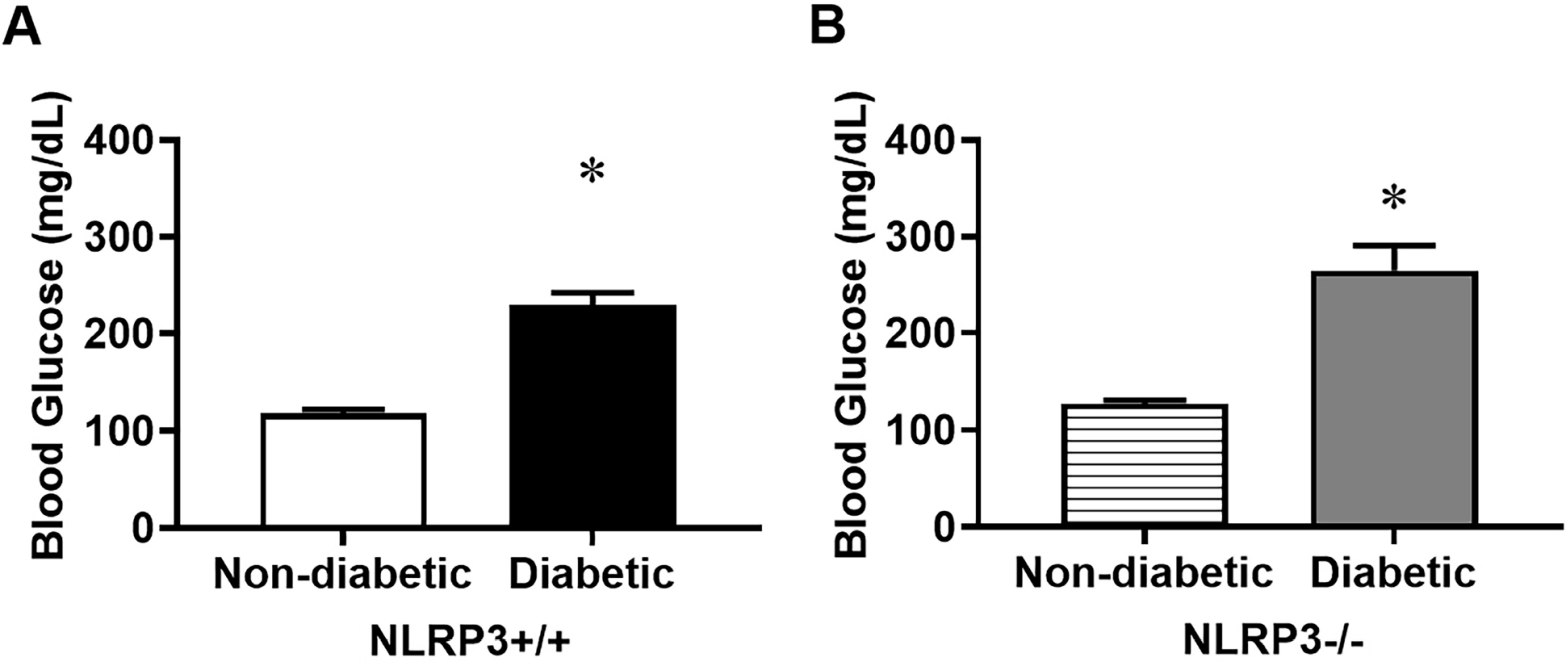
*NLRP3* gene deletion does not impact blood glucose of female Akita mice. Blood glucose was measured in all groups at 30 weeks of age. (A) Female diabetics demonstrate marked hyperglycemia compared to non-diabetic controls. (B) In the absence of the *NLRP3* gene, and therefore NLRP3-mediated inflammation, blood glucose remains significantly elevated. Data are presented as mean ± standard error of the mean. N = 10 per group; *p < 0.05, one-way analysis of variances (ANOVA) with Tukey post hoc tests comparing all four groups. NLRP3, nucleotide-binding domain, leucine-rich–containing family, pyrin domain–containing-3.

Consistent with prior studies [8], female diabetic Akita mice exhibit marked hyperglycemia by 30 weeks of age (Fig. 1A). Although *NLRP3* gene deletion has previously been demonstrated to prevent diabetic females from developing bladder inflammation [7,8], *NLRP3* gene deletion has no impact on blood glucose (Fig. 1B). This suggests hyperglycemia-induced inflammation is a key factor responsible for the development and progression of DBD in this model.

### 3.2 Diabetes Dysregulates the Release of PGE2, but not PGF2α, as a Consequence of NLRP3-Dependent Inflammation

**Fig. 2.**
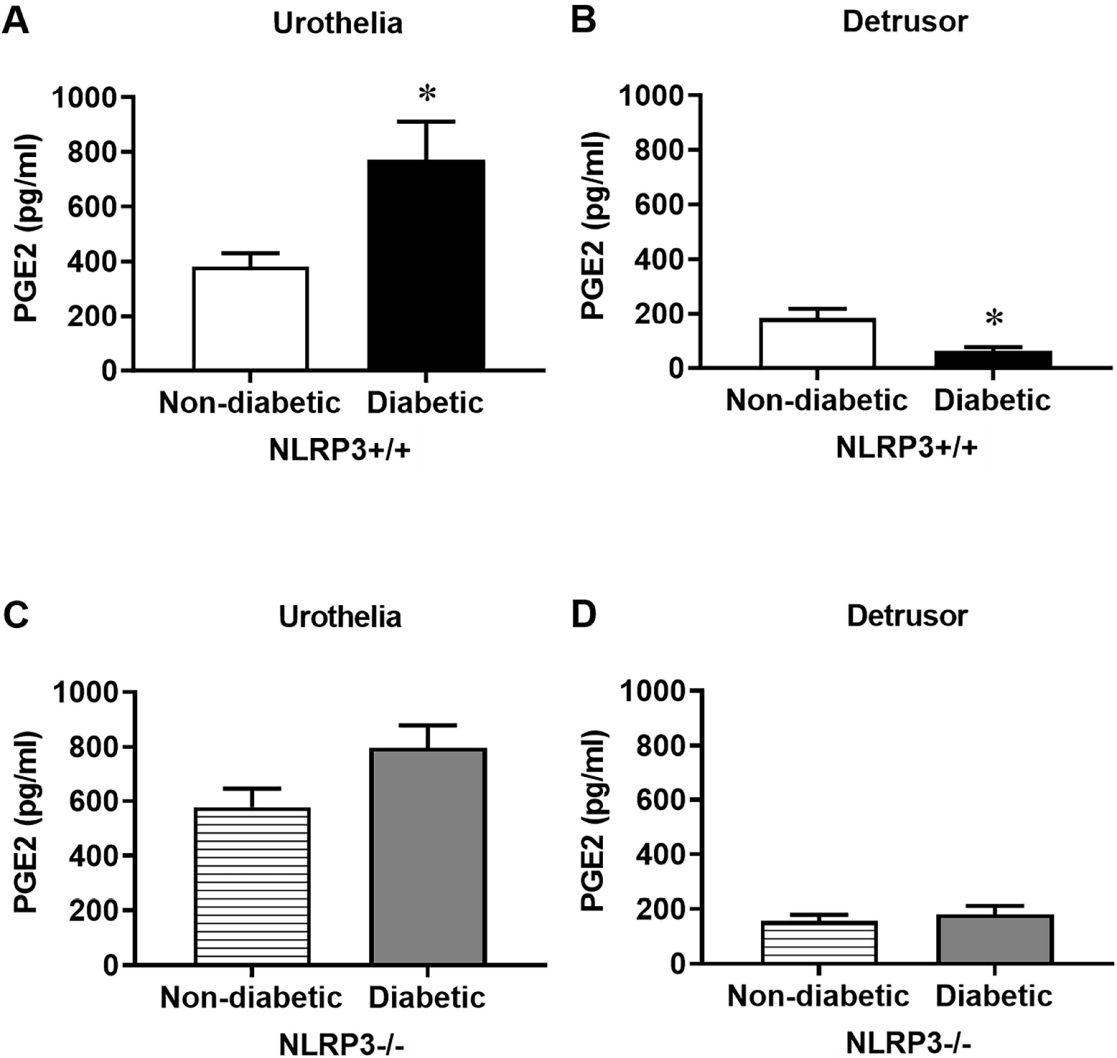
Diabetes increases prostaglandin E2 (PGE2) release from urothelia but conversely decreases PGE2 release from detrusors due to NLRP3-dependent inflammation. Strips of urothelia-lined mucosa and detrusors were stretched *ex vivo* to release PGE2, which was then quantified using an enzyme linked immunosorbent assay (ELISA). (A) Diabetes nearly doubles the amount of PGE2 released from urothelia. (B) However, in the detrusor, the amount of PGE2 released from detrusors is significantly reduced. (C) In diabetic mice lacking the *NLRP3* gene, no significant changes in PGE2 release are detected in either the urothelia or (D) detrusors. Data are presented as mean ± standard error of the mean. N = 4 per group, per tissue type; *p < 0.05, one-way ANOVA with Tukey post hoc tests comparing all four groups for each tissue type.

**Fig. 3.**
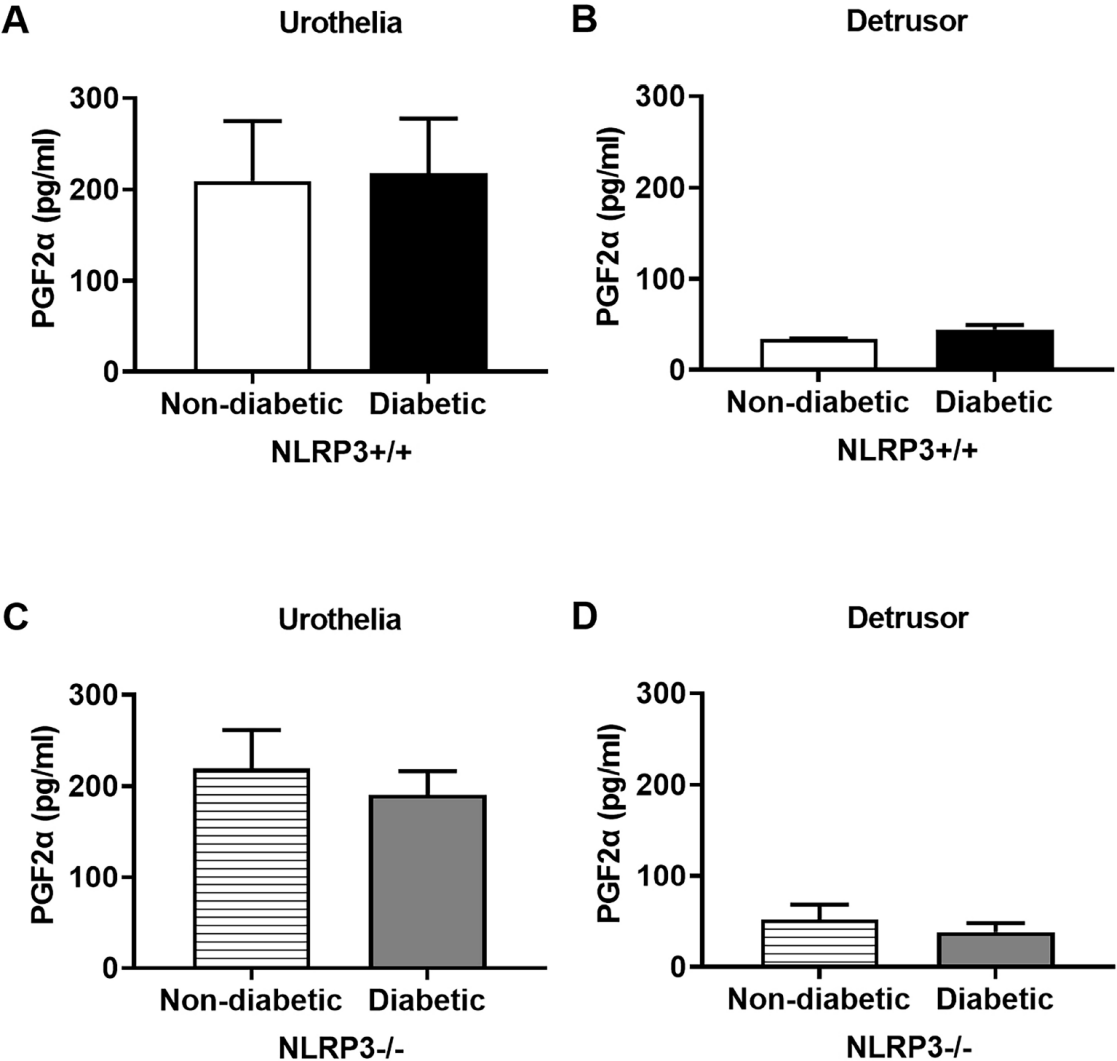
Prostaglandin F2*α* (PGF2*α*) release from urothelia and detrusors is not augmented by diabetes regardless of NLRP3. Strips of urothelia-lined mucosa and detrusors were stretched *ex vivo* to release PGF2*α*, which was then quantified using an ELISA. (A) PGF2*α* release does not differ between non-diabetic and diabetic urothelia and (B) detrusors. (C) Similarly, in the absence of *NLRP3*, PGF2*α* release is not impacted by diabetes in either urothelia or (D) detrusors. Data are presented as mean ± standard error of the mean. N = 4 per group, per tissue type; ANOVA with Tukey post hoc tests comparing all four groups for each tissue type.

The increased levels of prostaglandin metabolites found in the urine of patients with diabetes suggests diabetes increases prostaglandin production [13–15]; however, it is unclear where prostaglandin upregulation occurs – either systemically and/or locally within the bladder. In this set of experiments, we sought to determine if this increase may result from a change in its release from urothelia and/or detrusors. Diabetes increases the amount of PGE2 released from urothelia compared to non-diabetics (Fig. 2A). Surprisingly, diabetes dichotomously decreases the amount of PGE2 released from bladder smooth muscle (Fig. 2B). In order to determine if these changes were due to NLRP3-mediated inflammation, we performed the same experiments in diabetic females lacking the *NLRP3* gene. We have previously shown that diabetic mice lacking the *NLRP3* gene do not develop inflammation in the bladder, even at 30 weeks, and the mice are protected from developing UAB [7,8]. Here, deletion of the *NLRP3* gene also prevents diabetes from dysregulating PGE2 release in both urothelia and detrusors (Fig. 2C,D) as there are no significant differences in PGE2 release compared to non-diabetics without the *NLRP3* gene. The same pattern does not apply to the release of PGF2*α*. Interestingly, diabetes does not affect the amount of PGF2*α* released from urothelia and detrusors (Fig. 3A,B) regardless of the presence or absence (Fig. 3C,D) of the *NLRP3* gene.

### 3.3 Diabetes does not Impact Contractions Mediated by PGE2, but Increases Contractile Force Generated by FP Receptor Activation due to NLRP3-Dependent Inflammation

**Fig. 4.**
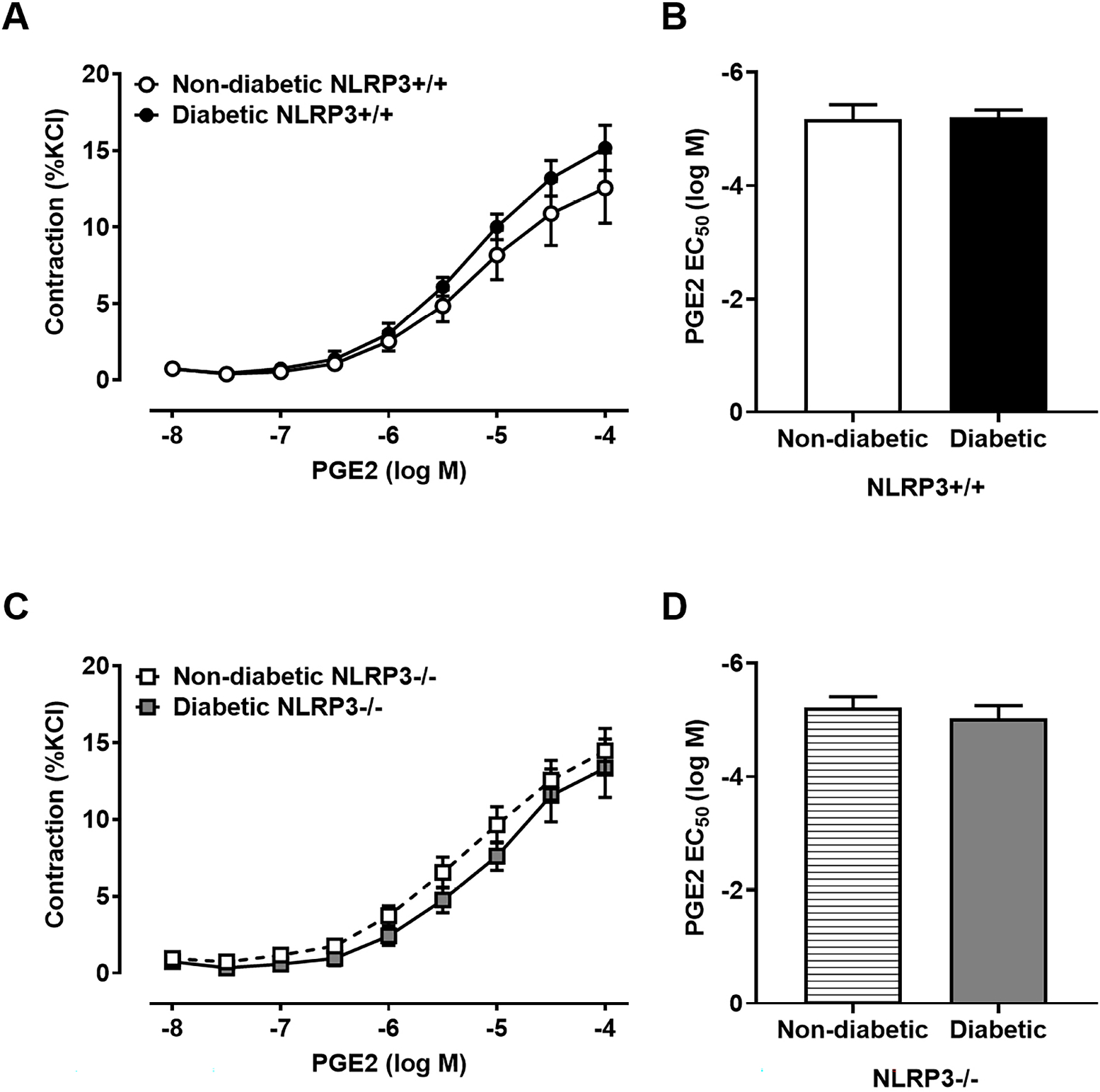
Bladder contractions mediated by PGE2 are not impacted by NLRP3-mediated inflammation attributed to diabetes. Strips of bladder tissue with intact mucosa and urothelia were used to assess smooth muscle function *ex vivo* in a myograph apparatus. Contractile force was measured in response to cumulative increases in concentrations of PGE2. (A) Contractile force generated in response to PGE2 and the (B) potency (EC_50_) of PGE2 does not differ between bladders from non-diabetic and diabetic groups in the presence or (C,D) absence of the *NLRP3* gene. Data are presented as mean ± standard error of the mean. N = 9 Non-diabetic *NLRP3*^+/+^, 10 Diabetic *NLRP3*^+/+^, 9 Non-diabetic *NLRP3*^−/−^, 7 Diabetic *NLRP3*^−/−^; two-way ANOVA with Bonferroni post hoc tests comparing all four groups. Concentration response curves were fitted to a logistic function by nonlinear regression and the −log half maximal effective concentration (EC_50_) was calculated.

**Fig. 5.**
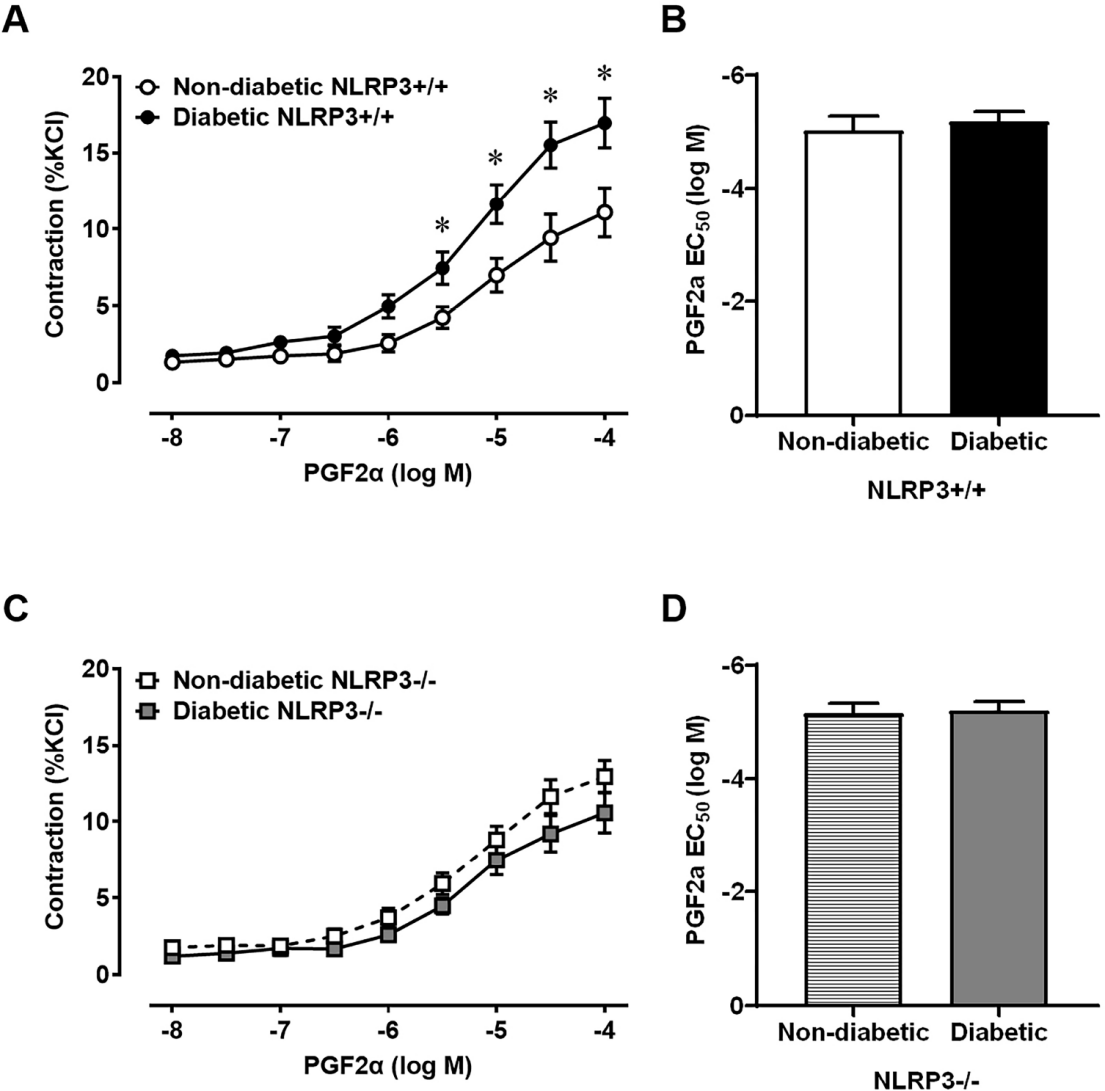
Diabetes increases PGF2*α*-mediated contractile force through NLRP3-dependent mechanisms. Strips of bladder tissue with intact mucosa and urothelia were used to assess smooth muscle function *ex vivo* in a myograph apparatus. Contractile force was measured in response to cumulative increases in concentrations of prostaglandin F2*α* (PGF2*α*). (A) Diabetic bladders demonstrate a significantly higher contractile force in response to PGF2*α* than non-diabetic controls. (B) However, the potency (EC_50_) of PGF2*α* does not significantly differ between groups. (C,D) In diabetic bladders lacking the *NLRP3* gene, no significant differences in PGF2*α*-mediated contractile force and potency (EC_50_) are noted between non-diabetic and diabetic groups. Data are presented as mean ± standard error of the mean. N = 9 Non-diabetic *NLRP3*^+/+^, 10 Diabetic *NLRP3*^+/+^, 8 Non-diabetic *NLRP3*^−/−^, 7 Diabetic *NLRP3*^−/−^; *p < 0.05, two-way ANOVA with Bonferroni post hoc tests comparing all four groups. Concentration response curves were fitted to a logistic function by nonlinear regression and the −log half maximal effective concentration (EC_50_) was calculated.

Prostaglandins exert physiological effects on tissues within limited proximity to the site from which they are released and, in the bladder, prostaglandins regulate detrusor contractility. To determine how prostaglandin-mediated contractile force is impacted by diabetes, *ex vivo* concentration response curves to PGE2 and PGF2*α* were constructed using strips of bladder with intact urothelia. Even though diabetes dysregulates the release of PGE2, the maximum contractile force generated by PGE2 is unaffected by diabetes (Fig. 4A). Further evaluation of the EC_50_ for PGE2 reveals no differences between the two groups, suggesting diabetes in this model does not impact the affinity EP receptors in the bladder (Fig. 4B). No differences in the PGE2-mediated maximal contractile force or EC_50_ are evident in diabetic mice lacking the *NLRP3* gene as well (Fig. 4C,D). Surprisingly, even though these bladders are from diabetic mice with a demonstrated UAB phenotype, they actually exhibit an increase in contractile force to PGF2*α* (Fig. 5A). Although contractile force increases, there is no significant difference in the PGF2*α* EC_50_ (Fig. 5B). The increased contractile force generated by PGF2*α* is absent in diabetic mice lacking *NLRP3* (Fig. 5C) and there are no significant differences in the EC_50_ values between diabetics and non-diabetics (Fig. 5D) – thus indicating a critical role of NLRP3-dependent inflammation in the dysregulation of PGF2*α*-mediated bladder contractions.

**Fig. 6.**
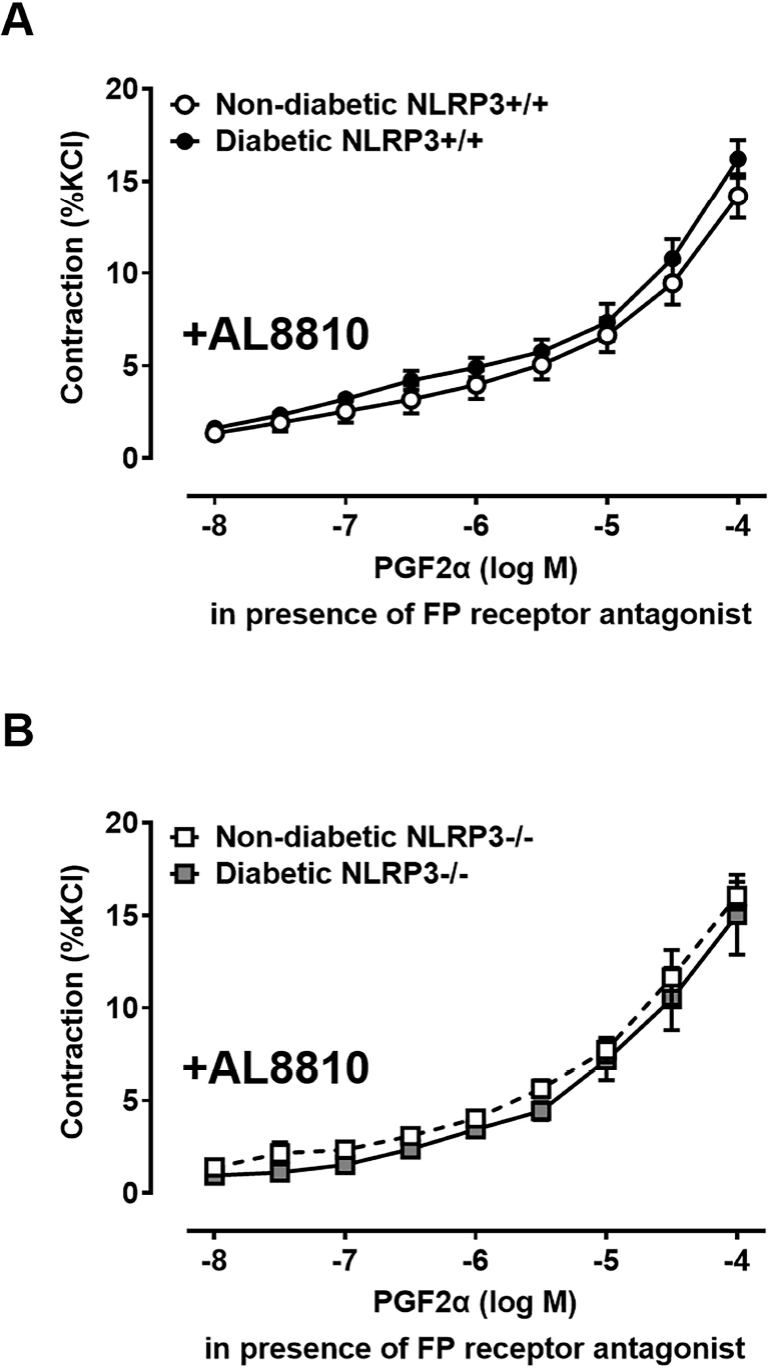
FP receptor activation facilitates the increase in PGF2*α*-mediated contractions observed in diabetic bladders. The same strips of bladder tissue shown in Fig. 5 were incubated in a partial FP receptor antagonist, AL8810, during evaluation of *ex vivo* smooth muscle function using a myograph apparatus. Following a brief incubation period, a concentration response curve to prostaglandin F2*α* (PGF2*α*) was repeated. (A) In the presence of AL8810, significant differences in PGF2*α*-mediated contractions are no longer observed in diabetic bladders as contractions are comparable to non-diabetic bladders. (B) Elimination of the *NLRP3* gene in conjunction with AL8810 causes no additional changes in contractile force mediated by PGF2*α*. Data are presented as mean ± standard error of the mean. N = 6 Non-diabetic *NLRP3*^+/+^, 8 Diabetic *NLRP3*^+/+^, 5 Non-diabetic *NLRP3*^−/−^, 7 Diabetic *NLRP3*^−/−^; two-way ANOVA with Bonferroni post hoc tests comparing all four groups.

Although PGF2*α* has a higher affinity for FP receptors, it can activate multiple prostaglandin receptor types. Correctly identifying the receptor population responsible for the increased PGF2*α* contractile force is necessary to identify potential therapeutic targets. To determine the contribution of FP receptor activation, PGF2*α* concentration response curves were repeated in the presence of a FP receptor antagonist, AL8810. In the presence of AL8810, contractile force generated by PGF2*α* no longer differs between diabetic and non-diabetic bladders (Fig. 6A). Therefore, FP receptor activation is responsible for the elevated contractile force of diabetic bladders in response to PGF2*α*. In the absence of NLRP3 inflammation, diabetic mice without the *NLRP3* gene do not develop an increased contractile response to PGF2*α*, and in the presence of an FP receptor agonist, this pattern remains as there are no significant differences in PGF2*α* contractions between both diabetic and non-diabetic mice lacking the *NLRP3* gene (Fig. 6B).

### 3.4 Diabetes Causes a Four-Fold Increase in FP_A_ Receptors within the Detrusor due to NLRP3

**Fig. 7.**
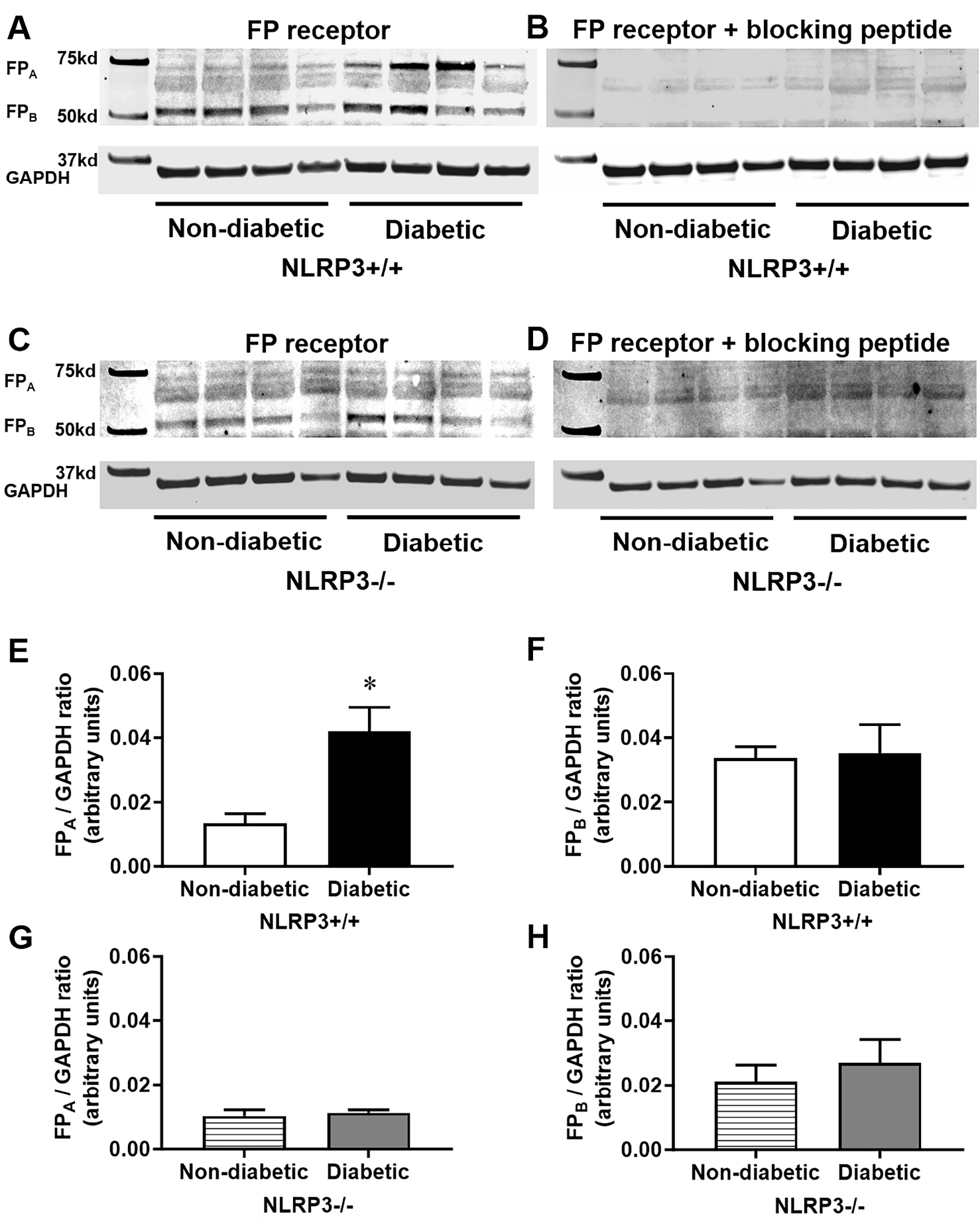
Diabetes increases FP_A_ receptor protein expression, but not FP_B_, in detrusors as a consequence of NLRP3. Western blots were performed to determine protein expression of the FP_A_ and FP_B_ receptor isoforms in detrusors from each group. An additional FP receptor blocking peptide was utilized as a control to ensure specific antibody binding. (A) Representative images show two distinct FP receptor isoforms between the molecular weights of 50–75 kd in both non-diabetic and diabetic detrusors with *NLRP3* and (C) in groups lacking the *NLRP3* gene as well as expression of the housekeeping protein GAPDH used for normalization of FP receptor expression. (B,D) When western blots are performed in the presence of a FP receptor blocking peptide, FP receptor isoforms are undetectable in all four groups. (E) Diabetes increases expression of the higher molecular weight FP_A_ isoform in detrusors by approximately 400% (F) while having no effect on the lower molecular weight FP_B_ isoform. (G) In the absence of *NLRP3*, diabetes has no effect on either the FP_A_ or (H) FP_B_ isoforms. Data are presented as mean ± standard error of the mean. N = 5–6 per group; *p < 0.05, one-way ANOVA with Tukey post hoc tests comparing all four groups.

FP receptors are highly expressed in human bladder smooth muscle [23]. Multiple FP receptor isoforms have been identified in mouse liver, spleen, kidneys, testes, and vas deferens [24]. The most abundant of which are the FP_A_ and FP_B_ isoforms. Structurally, the FP_A_ isoform is larger as the FP_B_ isoform has a substitution of the last 46 amino acids in the carboxyl terminus with a single amino acid [25]. Prior to this study, FP receptor expression had not been demonstrated in murine bladders. Our data shows two distinct FP receptor isoforms within the predicted molecular weight range in mouse detrusors from all mouse groups (Fig. 7A,C). Interestingly, diabetes causes a four-fold increase in the higher molecular weight FP_A_ receptor isoform (Fig. 7E) but does not impact protein expression of the lower weight FP_B_ isoform (Fig. 7F). To determine if these changes are dependent on the NLRP3 inflammasome, protein expression was measured in groups lacking the *NLRP3* gene. In the absence of *NLRP3*, diabetes does not impact expression of either FP receptor isoform (Fig. 7G,H). In order to confirm which western blot bands of detected proteins were specific FP receptor isoforms, a blocking peptide was used as a control. In the presence of a blocking peptide, both bands of FP receptor isoforms are undetectable (Fig. 7B,D), thereby validating the bands are the expected isoforms.

## 4. Discussion

DBD is a multifactorial pathology for which the precise mechanisms remain elusive. One well-regarded contributor to DBD is inflammation, which has been linked to aberrant prostaglandin synthesis and release [4,13–15]. However, a link between DBD, the contribution of specific inflammatory signaling pathways, and aberrant prostaglandin regulation in the bladder has not been established in current literature. Here, we demonstrate that diabetes-associated inflammation mediated by the NLRP3 inflammasome may be responsible for dysregulated prostaglandin release in the bladder, enhanced prostaglandin-mediated detrusor contractions, and increased prostaglandin receptor expression in the female Type 1 diabetic Akita model that develops UAB [8]. Our data suggests NLRP3 inhibition may prevent the development of DBD in part by preventing such dysregulation of prostaglandin signaling pathways. In the pursuit of elucidating these NLRP3-dependent mechanisms, we demonstrate an upregulation of FP receptors in diabetic bladders. Pharmacological agonists targeting these FP receptor populations may increase the contractile force of otherwise underactive bladders and possibly prove to be an effective therapeutic option to treat existing DBD.

Systemic low-grade inflammation is a well-appreciated characteristic of diabetes. Patients with diabetes and refractory overactive bladder (OAB) have been shown to have significant urothelial inflammation as indicated by an elevated number of activated mast cells [26]. Our laboratory has extensively investigated the critical role of the NLRP3 inflammasome in mediating urothelial inflammation [7,8,27–29]. NLRP3 is ubiquitously expressed in urothelia and can be activated by metabolites commonly found in urine of patients with diabetes [7]. Upon activation, NLRP3 causes urothelial cell death and releases pro-inflammatory cytokines which subsequently recruit mast cells to further perpetuate an inflammatory cascade. In female Akita mice, NLRP3 is a critical factor responsible for the development of both OAB and UAB. Genetic deletion of NLRP3 in female Akita mice prevents bladder inflammation assessed using an Evans blue assay and the development of either DBD phenotype [7,8]. This poses the novel possibility that NLRP3 inhibitors may prevent the development of DBD in human patients. Unfortunately, there are currently no clinical biomarkers to predict the development of DBD and patients generally do not seek urological consult until after symptoms of DBD have developed. Thus, in order to identify novel therapeutic targets to treat existing DBD, our efforts focused on elucidating the poorly understood NLRP3-dependent downstream signaling pathways which regulate bladder function.

NLRP3-dependent inflammation has been shown to facilitate murine proximal tubule cell injury by increasing cyclooxygenase-2-mediated PGE2 synthesis [10]. High levels of prostaglandin metabolites found in the urine of patients with diabetes suggest this signaling pathway may be consistent with pathology contributing to clinical DBD. Interestingly, inflammatory conditions are often associated with increased prostaglandin synthesis, and high levels of PGE2 and PGF2*α* metabolites in urine are regarded as an indirect measurement of systemic inflammation [4,13–15]. Preclinical studies using streptozotocin-induced Type 1 diabetic rats have investigated the impact of diabetes on the release of prostaglandins from bladder urothelia and detrusors [16]. They found basal levels of normalized PGE2 and PGF2*α* release were decreased in diabetic urothelia while diabetic detrusors demonstrated no change in PGE2 release and increased PGF2*α* release [16]. Although these findings are interesting, they represent basal prostaglandin release. As bladders fill with urine, they are constantly undergoing cycles of dilation and contraction. From a physiological perspective, it’s important to consider that stretch is a key mechanism facilitating prostaglandin release. Though direct comparisons cannot be made between the previous and current study, both provide insight into independent aspects of prostaglandin release from diabetic bladders. In the current study, we find PGE2 release from stretched diabetic urothelial to be elevated, but release from stretched detrusors is diminished. This dichotomous pattern of PGE2 release is particularly interesting since NLRP3 is highly expressed in urothelia but minimally expressed in detrusors [28] – suggesting an NLRP3-dependent mechanism of PGE2 production and release. Indeed, we investigate this possibility in diabetic mice lacking the *NLRP3* gene and demonstrate that PGE2 release is not elevated. A similar NLRP3-dependent dysregulation of PGF2*α* release was anticipated, but it is unclear why diabetes does not impact PGF2*α* release in this model. We hypothesize that multiple mechanisms responsible for prostaglandin release may be independently dysregulated by diabetes. In addition to bladder stretch, prostaglandins can be released from the bladder in response to neuropeptides such as bradykinin. In strips of bladder from streptozotocin-induced Type 1 diabetic rats, diabetes had no effect on the bradykinin-stimulated release of PGE2, but increased the bradykinin-stimulated release of PGF2*α* – a curiously inverse pattern of prostaglandin release from the current study, but one that demonstrated independent regulation of PGE2 and PGF2*α* release during diabetes [17].

Since prostaglandins exert physiological effects on tissues near the site of release before rapidly degrading, we postulated that changes in prostaglandin release would associate with changes in the prostaglandin-mediated contractile force of the detrusor. Multiple studies demonstrated prostaglandins directly contract detrusor *ex vivo* [16,19–21]. Given the significant increase in stretch-mediated PGE2 release, the lack of significant changes in PGE2-mediated contractile force in diabetic bladders was unanticipated. This could be due to changes in the expression of multiple forms of EP receptors in the detrusor. Changes in the expression of EP1 and EP3 receptors, which are pro-contractile, may be masked by changes in the expression of EP2 and EP2 receptors which facilitate relaxation [30]. However, since the potency of PGE2, indicated by the EC_50_, does not statistically differ between non-diabetic and diabetic groups, it is likely that EP receptor populations in the bladders of Akita mice are not impacted by diabetes. Regardless, the physiologic contractile response to PGE2 is not impacted by diabetes in the current study. Further classification of EP receptor expression in the bladder was therefore not a priority. In addition, clinical studies have evaluated intravesically administered PGE2 as a treatment for UAB due to other etiologies [31,32]. In each study, intravesical PGE2 was ineffective as a treatment for UAB, further supporting our findings and this decision.

Diabetes-associated pathological changes in the mechanisms by which prostaglandins generate contractile force in the bladder are poorly understood, particularly regarding PGF2*α*. Given the UAB phenotype of the female diabetic mice used in this study, one may reasonably expect a decrease in contractile force generated by PGF2*α*, especially considering a decrease in PGF2*α*-mediated force has been shown in streptozotocin-induced diabetic rats [16]. However, we provide evidence that diabetes actually increases contractile force generated in response to PGF2*α*, specifically by activation of detrusor-bound FP receptors. This is particularly remarkable given the potency of PGF2*α* is comparable between both groups. As perplexing as this finding was, we sought to further characterize the physiological basis for higher force generation due to FP receptor activation and hypothesized that it may likely be attributed to an increase in the number FP receptors in the detrusor. Consistent with other tissue [24], two isoforms of FP receptors are evident in detrusors. The four-fold increase in FP_*A*_ receptor expression and lack of changes in FP_*B*_ receptor expression strongly suggest the increased FP receptor-mediated contractile force in diabetic bladders is likely due to activation of FP_*A*_ receptors. Interestingly, in this animal model, the upregulation of FP receptor populations and increased force generated by FP receptor activation is driven by NLRP3-dependent inflammation associated with diabetes.

Inflammation mediated by NLRP3 is attributed to a host of negative pathologies contributing the development of DBD in female Akita mice [7,8]. However, the NLRP3-dependent increase in contractile force generated by FP receptor activation as well as the increased production of urothelial PGE2 appear more likely to be compensatory mechanisms rather than causes of UAB in diabetics. In an upcoming study, we will investigate the nature of prostaglandin synthesis and receptor expression at an earlier time point when the Akita develops overactive bladder. We have previously demonstrated that female Akita mice demonstrate a progression from OAB at 15 weeks of age to UAB by 30 weeks of age [7,8]. If we see results similar to what is observed at 30 weeks, that would suggest that this is a compensatory mechanism that is overwhelmed after a further 15 weeks of disease progression.

Regardless of the reasons why FP receptors are upregulated, our data suggests FP receptor agonists may be effective therapies to treat existing underactive DBD. This is interesting given the availability of FP receptor agonists currently being used to treat non-urological conditions such as glaucoma. One commonly prescribed FP receptor agonist is latanoprost. This medication is available as an ophthalmic solution which reaches a peak systemic half-life in 5 minutes and has an elimination half-life of only 17 minutes [33]. Despite this short half-life and low potential for systemic effects, OAB symptoms can develop as a consequence of latanoprost therapy. In one case report, a 62-year-old female patient began voiding small amounts of urine at an increased frequency and exhibited urge incontinence upon taking latanoprost [34]. Conventional challenge-rechallenge testing confirmed latanoprost was the cause of her OAB symptoms, which subsided upon terminating latanoprost therapy. Furthermore, similar to the *ex vivo* PGF2*α*-mediated bladder contractions reported in this study, both PGF2*α* and latanoprost have also been shown to directly stimulate bladder contractions *ex vivo* in rats, macaques, and humans [20]. Future investigations will determine the effectiveness of FP receptor agonists in the treatment of existing DBD.

## 5. Conclusion

NLRP3-dependent inflammation associated with diabetes dysregulates the release of prostaglandins and increases the capacity of underactive bladders to generate contractile force in response to prostaglandins that activate FP receptors within the detrusors due, in part, to a fourfold upregulation of FP receptor protein expression. These novel findings suggest FP receptor agonists –of which there are commercially available examples [34] – may be utilized as a novel therapeutic to treat existing underactive DBD.

## Supplementary Material

Supplemental Data

## Data Availability

Uncropped western blot images used for analysis are provided as Supplementary Figs. 1, 2, and 3. All raw numerical data used for analysis and reported within figure graphs are provided as Supplementary Tables 1, 2, 3, 4.1, 4.2, 5.1, 5.2, 6, and 7.
