# Supplementary material for: Female Type 1 Diabetic Akita Mice Demonstrate Increased Bladder Contractility via FP Receptor Activation due to NLRP3-Mediated Inflammation
Source: Front Biosci (Landmark Ed). Author manuscript; Available in PMC 2025 Apr 18. (PMC11372815; doi:10.31083/j.fbl2904154)
Supplement: Supplemental Data [file NIHMS2015504-supplement-Supplemental_Data.pdf]

Supplemental Figure 1

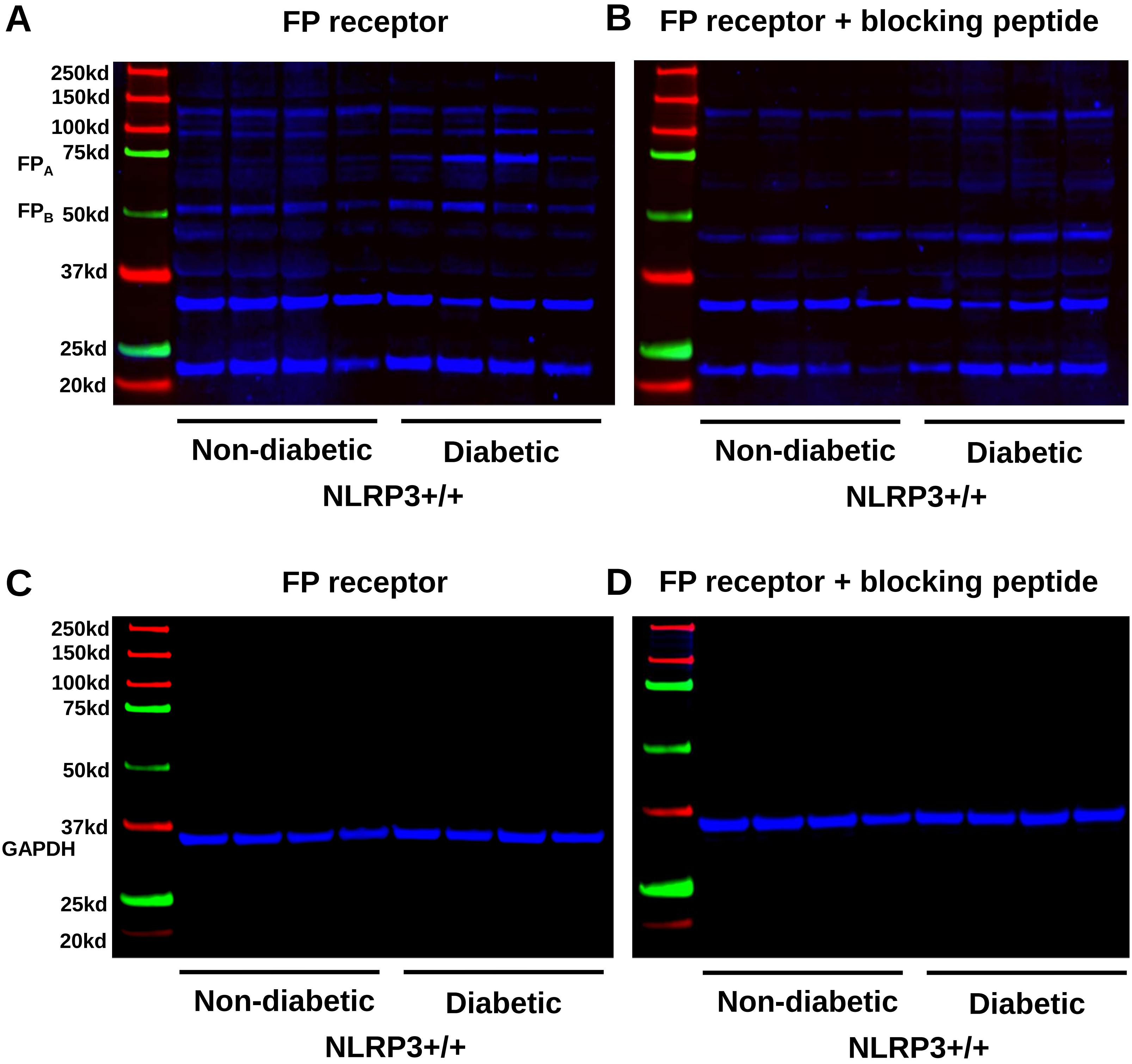

Supplemental Figure 1: FP receptor expression in NLRP3+/+ mice. Protein expression of FP receptor isoforms was determined using western blots as described in the methods section. **A)** In NLRP3+/+ non-diabetic and diabetic bladders, two distinct FP receptor isoforms, FP_A and FP_B, are evident in the 50-67 kDa target range set forth by the antibody manufacturer. **B)** However, in the presence of a FP receptor blocking peptide, these two isoforms are not detected – thereby validating the specificity of the antibody for FP receptors. **C and D)** Protein expression of GAPDH was measured on both membranes and used to normalize expression of FP receptor populations. N=4 Non-diabetic NLRP3+/+, 4 diabetic NLRP3+/+.

Supplemental Figure 2

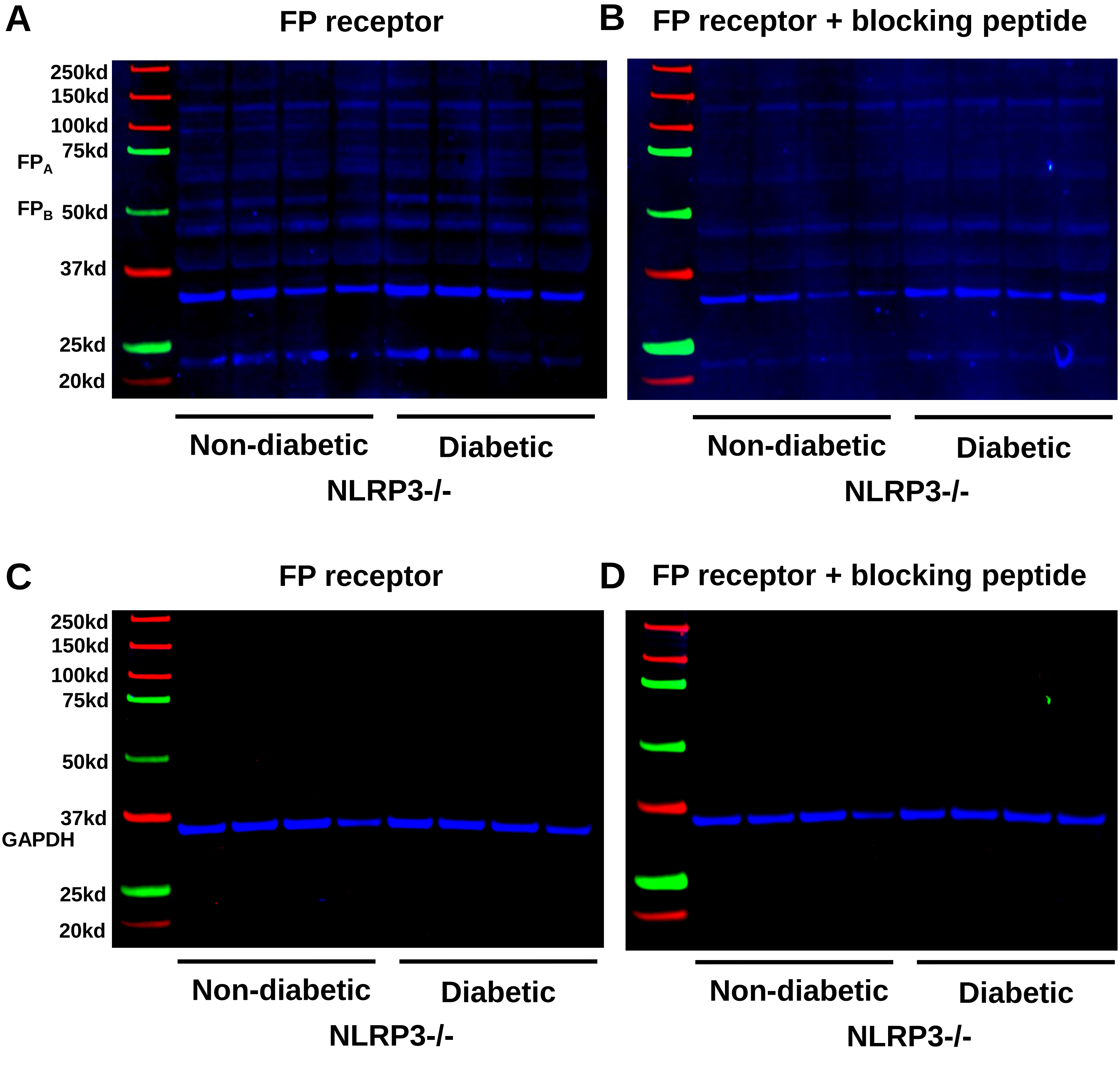

Supplemental Figure 2: FP receptor expression in NLRP3-/- mice. Protein expression of FP receptor isoforms was determined using western blots as described in the methods section. **A)** In NLRP3-/- non-diabetic and diabetic bladders, two distinct FP receptor isoforms, FP$_A$ and FP$_B$, are evident in the 50-67 kDa target range set forth by the antibody manufacturer. **B)** However, in the presence of a FP receptor blocking peptide, these two isoforms are not detected – thereby validating the specificity of the antibody for FP receptors. **C and D)** Protein expression of GAPDH was measured on both membranes and used to normalize expression of FP receptor populations. N=4 Non-diabetic NLRP3-/-, 4 diabetic NLRP3-/-.

Supplemental Figure 3

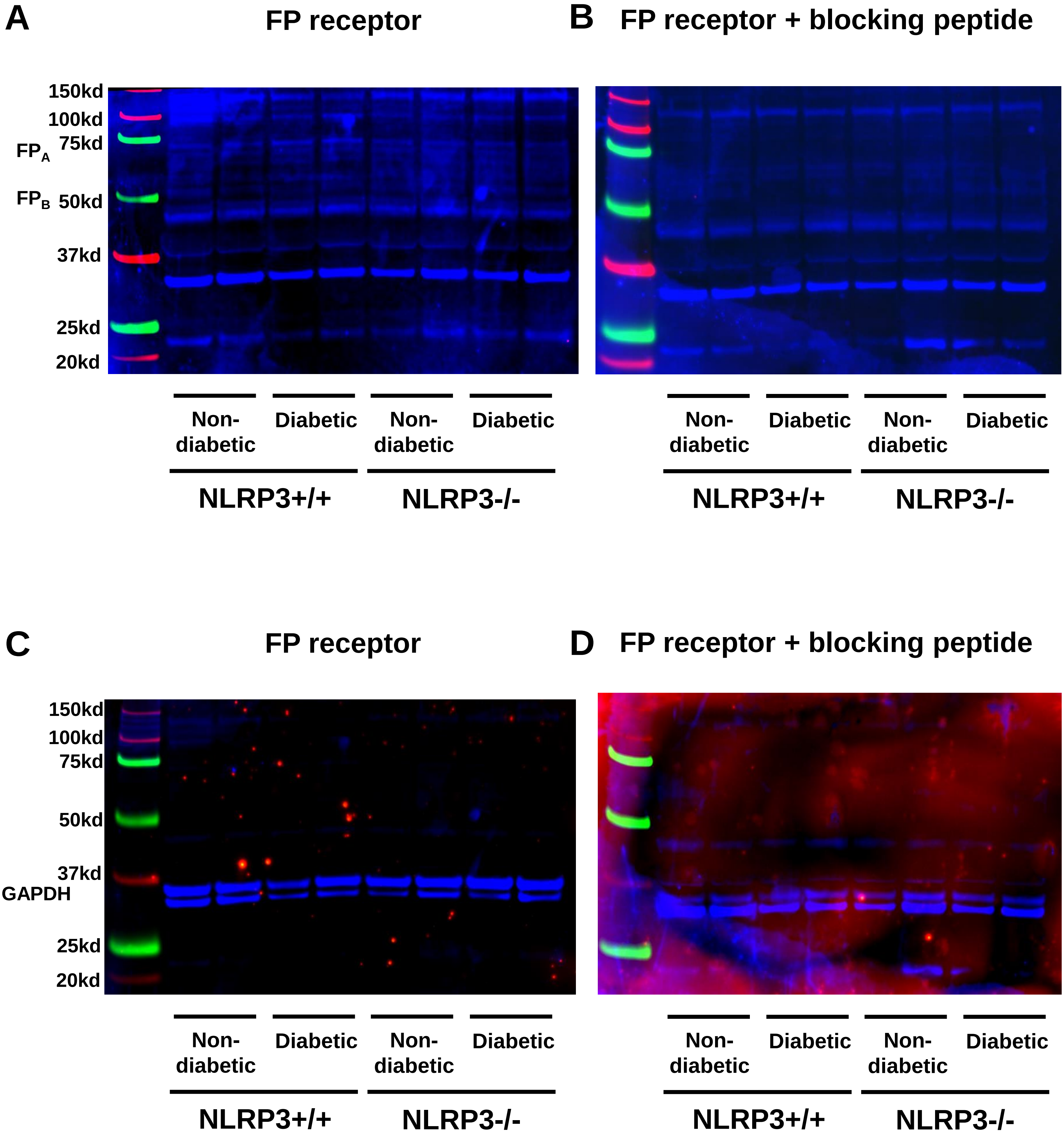

Supplemental Figure 3: FP receptor expression in Non-diabetic and Diabetic mice with and without the NLRP3 gene. Protein expression of FP receptor isoforms was determined using western blots as described in the methods section. **A)** In all groups, two distinct FP receptor isoforms, FP_A and FP_B, are evident in the 50-67 kDa target range set forth by the antibody manufacturer. **B)** However, in the presence of a FP receptor blocking peptide, these two isoforms are not detected – thereby validating the specificity of the antibody for FP receptors. **C and D)** Protein expression of GAPDH was measured on both membranes and used to normalize expression of FP receptor populations. N=2 Non-diabetic NLRP3+/+, 2 diabetic NLRP3+/+. 2 Non-diabetic NLRP3-/-, 2 diabetic NLRP3-/-.

Supplemental Table 1

Blood Glucose (mg/dL)			
Non-diabetic NLRP3+/+	Diabetic NLRP3+/+	Non-diabetic NLRP3-/-	Diabetic NLRP3-/-
102	209	126	278
94	221	139	246
107	284	130	322
137	201	133	259
118	221	109	295
124	204	129	223
129	195	116	449
123	272	126	148
123	203	112	203
119	296	147	228

Supplemental Table 1: Raw data for Figure 1. The blood glucose (mg/dL) of all four groups of mice represented in Figure 1 is provided. Each data cell represents an individual n.

Supplemental Table 2

PGE2 (pg/ml) Urothelia			
Non-diabetic NLRP3+/+	Diabetic NLRP3+/+	Non-diabetic NLRP3-/-	Diabetic NLRP3-/-
325.0882	698.5103	741.4765	895.7451
282.2008	1154.986	454.7144	571.4205
490.9137	743.6356	633.9009	783.1954
431.0931	483.7189	481.3957	936.9858
PGE2 (pg/ml) Detrusor			
Non-diabetic NLRP3+/+	Diabetic NLRP3+/+	Non-diabetic NLRP3-/-	Diabetic NLRP3-/-
102.1038	69.95365	182.0781	241.2111
165.0827	20.56502	97.15585	175.0778
260.2563	85.50876	142.5793	95.83495
211.9188	73.32546	201.7309	210.823

Supplemental Table 2: Raw data for Figure 2. The amount of PGE2 released (pg/ml) from the urothelia and detrusors of all four groups of mice represented in Figure 2 is provided. Each data cell represents an individual n.

Supplemental Table 3

PGF2α (pg/ml) Urothelia			
Non-diabetic NLRP3+/+	Diabetic NLRP3+/+	Non-diabetic NLRP3-/-	Diabetic NLRP3-/-
107.0394	178.7370	325.6601	168.8308
138.7300	365.1836	136.1726	129.2310
397.8476	246.6236	168.5463	244.2723
194.3773	80.9368	246.3283	220.0876
PGF2α (pg/ml) Detrusor			
Non-diabetic NLRP3+/+	Diabetic NLRP3+/+	Non-diabetic NLRP3-/-	Diabetic NLRP3-/-
33.8690	52.5878	96.2463	34.4864
34.6515	42.7630	20.0228	29.2517
34.1583	50.3094	38.7817	22.4435
45.4164	30.4875	52.5330	66.5739

Supplemental Table 3: Raw data for Figure 3. The amount of PGF2α released (pg/ml) from the urothelia and detrusors of all four groups of mice represented in Figure 3 is provided. Each data cell represents an individual n.

Supplemental Table 4.1

PGE2 (%KCl) Non-diabetic NLRP3+/+										
**Concentration (log M)**										
**-8.0**	0.0000	0.6289	1.3272	0.7242	0.5596	0.3151	1.0378	0.8968	1.0937	
**-7.5**	0.0000	0.0000	0.6017	0.5657	0.0992	0.0000	0.6747	0.7139	0.7457	
**-7.0**	0.0000	0.0000	0.3115	1.9915	0.4061	0.0000	0.3117	0.9577	0.6761	
**-6.5**	0.3260	0.0000	0.9645	3.2589	1.0200	0.7392	0.8200	1.1406	1.1633	
**-6.0**	0.5332	0.0000	3.2136	6.6913	1.8640	2.1815	2.1995	2.5424	3.4602	
**-5.5**	1.8284	1.5540	7.7845	11.6551	3.0916	3.1147	4.5955	5.1632	4.9219	
**-5.0**	3.7970	4.4889	12.2103	19.3648	5.7004	5.7447	7.9354	7.3574	7.0100	
**-4.5**	6.1282	7.2005	17.2890	25.1735	6.8514	8.2050	9.3875	9.1249	8.6805	
**-4.0**	7.5270	10.2310	20.1186	27.9722	8.6928	8.7989	10.4040	9.7344	9.5157	

PGE2 (%KCl) Diabetic NLRP3+/+										
**Concentration (log M)**										
**-8.0**	1.0061	1.5693	0.6762	1.6812	0.4832	0.6818	0.4557	0.3070	0.0000	0.0000
**-7.5**	0.7033	1.5965	0.6762	0.4553	0.1687	0.6818	0.0000	0.0000	0.0000	0.0000
**-7.0**	1.6115	2.6569	1.7677	0.4553	0.0000	0.6116	0.1615	0.0000	0.0000	0.0000
**-6.5**	2.9738	4.7778	2.9319	0.7004	0.2080	1.4539	0.3576	0.0000	0.0000	0.0000
**-6.0**	4.5632	7.2250	5.4059	2.1716	1.9767	3.3490	2.6133	0.7186	1.7865	0.2423
**-5.5**	6.4553	10.1344	8.6803	5.1138	5.1996	6.2970	5.4574	3.9655	5.4140	4.2874
**-5.0**	10.3151	16.1979	12.4641	7.4430	9.4444	9.5959	9.0371	7.4867	8.1926	10.0289
**-4.5**	13.7966	22.1255	15.0109	9.6497	12.9817	13.9476	11.0476	9.5904	10.1607	13.6173
**-4.0**	16.6725	26.6936	17.6304	11.3660	13.2962	15.4918	12.3716	11.0080	11.6271	15.5746

PGE2 (%KCl) Non-diabetic NLRP3-/-										
**Concentration (log M)**										
**-8.0**	0.0853	1.7610	1.3680	0.3540	0.9375	0.7272	1.0529	1.1937	1.0287	
**-7.5**	0.0000	1.1891	1.1160	0.5649	0.6310	0.5410	0.0127	1.4500	1.0903	
**-7.0**	0.0000	1.8881	1.9440	0.5649	1.6118	0.8514	0.0000	2.6799	1.0287	
**-6.5**	0.2275	0.9985	1.6200	1.0921	2.7152	2.1552	0.3594	4.0124	2.6290	
**-6.0**	0.6257	2.2694	4.3561	1.8829	5.1673	5.1352	2.6565	6.7285	4.7217	
**-5.5**	2.7301	3.5403	7.1642	2.3047	7.8645	9.0465	6.6872	10.8796	8.6608	
**-5.0**	4.6640	6.2727	11.8083	4.9936	11.0522	12.3370	9.4177	14.4157	11.8614	
**-4.5**	8.4748	9.2592	14.5443	5.6262	14.8528	14.6341	12.7116	17.6444	15.1851	
**-4.0**	10.0674	13.1989	18.8644	5.8371	16.1402	16.2483	13.5784	19.6943	16.5391	

PGE2 (%KCl) Diabetic NLRP3-/-										
**Concentration (log M)**										
**-8.0**	1.5499	0.6023	0.3665	1.5717	1.1034	0.0000	0.0000			
**-7.5**	1.5499	0.2567	0.4926	0.0496	0.0000	0.0000	0.0000			
**-7.0**	2.4819	0.8097	0.7450	0.0000	0.0000	0.0000	0.0093			
**-6.5**	3.4986	0.9479	1.5441	0.5569	0.0000	0.0000	0.1764			
**-6.0**	5.4472	3.1598	3.1422	1.9099	1.2300	1.0337	0.9698			
**-5.5**	7.9890	5.5100	6.5489	5.2923	3.3827	2.1340	2.4313			
**-5.0**	10.9543	8.4822	9.5349	7.9983	7.4348	3.9678	4.8951			
**-4.5**	17.3934	16.7078	11.4275	13.4101	9.5875	6.2417	6.1896			
**-4.0**	20.1893	17.7446	12.6051	16.4543	11.4869	8.2222	6.6072			

Supplemental Table 4.1: Raw data for Figure 4. The raw data for each PGE2 concentration response curve (%KCl) from all 4 groups represented in Figure 4 is provided here. Each column of data represents an individual n.

Supplemental Table 4.2

PGE2 EC50 (log M)				
	Non-diabetic NLRP3+/+	Diabetic NLRP3+/+	Non-diabetic NLRP3-/-	Diabetic NLRP3-/-
log EC50	-5.177	-5.204	-5.217	-5.026
Standard Error	0.2518	0.131	0.1941	0.2306
n	9	10	9	7

Supplemental Table 4.2: Raw data for Figure 4. The raw values for the calculated PGE2 EC50 (log M) of each group represented in Figure 4 are provided here.

Supplemental Table 5.1

PGF2α (%KCl) Non-diabetic NLRP3+/+										
**Concentration (log M)**										
**-8.0**	1.3621	2.4472	0.9645	2.2555	0.6363	0.6544	1.6912	1.3844	0.6065	
**-7.5**	1.2067	2.2558	1.6900	3.2061	1.0967	0.5696	2.1269	1.2625	0.3281	
**-7.0**	1.4139	2.4153	2.4155	4.4206	0.7898	0.5696	1.4008	1.8110	0.3281	
**-6.5**	1.6212	2.0325	2.9234	5.0543	0.9432	0.1454	1.8364	2.1767	0.1889	
**-6.0**	2.0874	3.5000	3.7941	5.8464	1.0967	0.7392	2.4173	3.0909	0.4673	
**-5.5**	3.4344	5.6054	6.0432	7.8002	2.6313	3.8783	2.9255	4.9804	0.7457	
**-5.0**	5.8174	8.5722	9.3807	13.6089	4.4728	7.3566	4.5229	6.9307	2.2770	
**-4.5**	7.3716	9.9758	13.5162	18.9423	6.9281	9.1382	6.4106	9.6735	2.9034	
**-4.0**	8.6149	13.1978	15.5477	20.8962	7.4652	11.0895	8.4436	9.7953	4.8523	

PGF2α (%KCl) Diabetic NLRP3+/+										
**Concentration (log M)**										
**-8.0**	1.2331	1.8684	2.3498	1.1908	0.9548	0.8222	1.2893	2.0448	1.7479	3.8959
**-7.5**	2.2927	2.1947	2.2770	0.8230	1.7409	1.9452	1.0932	0.9930	1.9409	4.0917
**-7.0**	4.0334	4.4243	2.7136	0.5779	2.7235	2.4365	1.3384	2.1820	2.4811	3.5045
**-6.5**	5.1687	6.4636	2.6409	0.3327	3.1165	2.5067	1.3874	1.6332	2.8284	4.1569
**-6.0**	6.9851	9.7537	3.3685	1.0682	4.5314	5.1037	4.5257	3.3710	4.2949	6.6362
**-5.5**	10.6179	13.6420	5.0421	2.5393	7.2827	6.8585	5.3594	5.2917	6.8033	11.0075
**-5.0**	15.0075	20.4669	8.3893	5.8493	10.8593	11.0698	11.2928	9.2245	10.4694	13.6825
**-4.5**	18.1105	24.4096	9.8446	8.6689	15.3006	16.3341	16.1474	12.6543	13.2479	20.3374
**-4.0**	19.8512	26.8295	11.2999	10.9982	16.2439	19.3522	14.3331	13.7061	14.2899	22.4252

PGF2α (%KCl) Non-diabetic NLRP3-/-										
**Concentration (log M)**										
**-8.0**	1.1743	1.7610	1.1975	0.6310	3.5831	0.0560	2.2699	3.3676		
**-7.5**	1.5156	2.2694	2.2520	0.9375	2.0931	0.0000	3.1412	3.0598		
**-7.0**	1.8853	2.1423	2.0938	0.7536	2.3414	0.0000	2.2699	3.4907		
**-6.5**	2.4541	2.7777	3.4646	1.3053	2.4035	0.0000	3.9611	3.6753		
**-6.0**	4.0467	4.3028	4.2027	2.3474	4.2660	0.0000	5.2423	5.2141		
**-5.5**	5.4971	6.2091	6.5225	5.9642	7.6807	1.3563	7.3435	6.7528		
**-5.0**	7.9997	8.8780	8.8423	9.0292	11.0332	3.1766	10.7771	10.6920		
**-4.5**	10.5877	12.8812	9.3168	12.5234	14.8824	5.2569	13.8008	13.7694		
**-4.0**	12.4362	13.7708	10.8458	14.1785	15.2550	6.7305	14.4157	15.8621		

PGF2α (%KCl) Diabetic NLRP3-/-										
**Concentration (log M)**										
**-8.0**	1.2110	0.5332	0.0000	2.9246	1.7365	1.0484	0.8650			
**-7.5**	1.6346	0.8788	0.0000	2.9246	1.9898	0.9017	1.3661			
**-7.0**	1.4652	1.5700	0.1658	4.1085	1.9898	1.1218	1.4914			
**-6.5**	1.5499	1.8465	0.1658	3.7702	1.4833	0.9751	1.9089			
**-6.0**	3.7527	2.5377	1.1751	3.4320	1.9898	1.7819	3.4123			
**-5.5**	5.7014	5.9247	2.7733	4.1085	2.4963	4.7160	5.6255			
**-5.0**	10.1071	10.5559	5.4650	4.7850	4.5224	8.2369	8.6738			
**-4.5**	12.4794	12.0075	7.0631	5.4615	5.2821	11.6111	10.4695			
**-4.0**	13.3266	13.9429	8.8716	5.9688	6.5484	14.1051	11.2211			

Supplemental Table 5.1: Raw data for Figure 5. The raw data for each PGF2α concentration response curve (%KCl) from all 4 groups represented in Figure 5 is provided here. Each column of data represents an individual n.

Supplemental Table 5.2

PGF2α EC50 (log M)				
	Non-diabetic NLRP3+/+	Diabetic NLRP3+/+	Non-diabetic NLRP3-/-	Diabetic NLRP3-/-
log EC50	-5.019	-5.178	-5.166	-5.204
Standard Error	0.2534	0.1764	0.1656	0.1645
n	9	10	8	7

Supplemental Table 5.2: Raw data for Figure 5. The raw values for the calculated PGF2α EC50 (log M) of each group represented in Figure 5 are provided here.

Supplemental Table 6

PGF2α in the presence of AL8810 (%KCl) Non-diabetic NLRP3+/+								
**Concentration (log M)**								
**-8.0**	1.9077	1.8859	1.4036	0.9938	1.2556	0.6530		
**-7.5**	2.4155	4.0510	1.4036	0.8241	1.7638	1.0796		
**-7.0**	3.9392	4.7903	1.4803	1.5028	2.1995	1.3234		
**-6.5**	4.5921	6.2688	1.8640	1.9270	2.7077	1.6891		
**-6.0**	5.4628	7.0081	2.0942	2.7754	3.7242	2.7862		
**-5.5**	6.5511	8.2755	3.7055	4.0479	4.5229	3.2738		
**-5.0**	7.4217	10.8102	5.0099	5.4902	6.4106	4.7366		
**-4.5**	10.0337	14.8763	7.4652	8.7989	7.4997	8.0888		
**-4.0**	16.2007	19.2592	11.6085	12.4469	12.9452	12.6600		

PGF2α in the presence of AL8810 (%KCl) Diabetic NLRP3+/+								
**Concentration (log M)**								
**-8.0**	2.0587	1.9264	1.1513	0.7520	0.6028	2.0448	1.6321	2.8520
**-7.5**	2.6409	2.5393	1.7409	1.1731	1.0932	2.5936	2.7898	4.0917
**-7.0**	4.1689	4.0104	2.2125	1.8750	1.7797	3.0509	3.2529	5.2661
**-6.5**	5.2604	4.9912	2.9986	2.2962	2.4172	5.3831	4.0633	6.1795
**-6.0**	6.1336	5.8493	3.2737	4.1211	2.7604	5.4746	4.4878	7.0929
**-5.5**	7.8799	7.0752	3.7454	3.6298	4.3786	6.1606	5.0281	8.2020
**-5.0**	9.0442	8.5463	4.5314	4.2615	5.1142	8.4014	6.2630	12.5734
**-4.5**	11.2999	11.7337	7.1648	8.8238	8.9390	11.5568	9.8520	16.9447
**-4.0**	16.3206	15.7793	12.1957	14.5793	16.0493	16.9530	15.4862	22.3599

PGF2α in the presence of AL8810 (%KCl) Non-diabetic NLRP3-/-								
**Concentration (log M)**								
**-8.0**	1.1214	1.4279	1.6597	1.1425	1.5827			
**-7.5**	1.6118	1.1175	1.3563	2.3724	4.3524			
**-7.0**	2.2861	1.4279	2.0064	3.6024	2.3828			
**-6.5**	3.3895	2.2350	3.3066	3.7561	2.7521			
**-6.0**	4.2477	3.1042	4.7369	4.6274	3.4291			
**-5.5**	6.6998	5.9601	5.7770	5.9086	3.8600			
**-5.0**	8.9066	9.3747	7.0339	7.7022	5.6449			
**-4.5**	13.3816	16.5765	8.9409	10.6234	8.7224			
**-4.0**	17.3662	17.6319	13.4050	15.9019	15.9852			

PGF2α in the presence of AL8810 (%KCl) Diabetic NLRP3-/-								
**Concentration (log M)**								
**-8.0**	1.3804	2.3995	0.3665	1.2334	1.3566	0.0000	0.0000	
**-7.5**	2.0582	2.5377	0.4926	1.7408	0.9768	0.0000	0.0000	
**-7.0**	2.0582	3.0216	1.0814	1.2334	1.7365	1.0747	0.5403	
**-6.5**	3.2444	3.5746	1.5020	1.5717	2.3697	2.7618	1.6260	
**-6.0**	4.4305	3.7819	2.0908	3.4320	2.4963	4.2288	3.6304	
**-5.5**	5.0236	5.3026	3.0161	3.7702	2.7496	6.0626	5.1337	
**-5.0**	7.9890	9.2426	3.7310	5.4615	3.6360	10.5371	9.7272	
**-4.5**	11.8863	15.1871	5.1610	6.8144	5.7886	15.3783	13.1931	
**-4.0**	15.6142	21.4081	10.0817	9.8586	8.7011	22.8602	16.7008	

Supplemental Table 6: Raw data for Figure 6. The raw data for each PGF2α in the presence of AL8810 concentration response curve (%KCl) from all 4 groups represented in Figure 6 is provided here. Each column of data represents an individual n.

Supplemental Table 7

FPA / GAPDH ratio (arbitrary units)			
Non-diabetic NLRP3+/+	Diabetic NLRP3+/+	Non-diabetic NLRP3-/-	Diabetic NLRP3-/-
0.006106	0.058306	0.007439	0.008388
0.008145	0.031955	0.007440	0.010698
0.012022	0.022598	0.003909	0.010980
0.010774	0.049289	0.012976	0.015417
0.014611	0.066934	0.017307	0.011350
0.028266	0.022020	0.012249	0.010558
**FPB / GAPDH ratio (arbitrary units)**			
Non-diabetic NLRP3+/+	Diabetic NLRP3+/+	Non-diabetic NLRP3-/-	Diabetic NLRP3-/-
0.036226	0.018815	0.026585	0.042592
0.041211	0.001064	0.033996	0.03634
0.04425	0.057575	0.022695	0.026568
0.033098	0.057779	0.019242	0.029178
0.024191	0.039369	0.00304	0.000479
0.023376	0.036593		

Supplemental Table 7: Raw data for Figure 7. The raw data for the FPA / GAPDH ratios and FPB / GAPDH ratios (arbitrary units) obtained via western blotting in Figure 7 is provided here. Each data cell represents an individual n.
